# Diagnostic Delays and Treatment Implications for Patients with Isoniazid-Resistant Tuberculosis: A Case Report and Review of the Literature

**DOI:** 10.1093/ofid/ofz222

**Published:** 2019-05-14

**Authors:** Gregory Olson, Ruvandhi R Nathavitharana, Philip A Lederer

**Affiliations:** 1 Section of Infectious Diseases and Global Health, University of Chicago Pritzker School of Medicine, Chicago, Illinois; 2 Division of Infectious Diseases, Department of Medicine, Beth Israel Deaconess Medical Center and Harvard Medical School, Boston, Massachusetts; 3 Section of Infectious Diseases, Department of Medicine, Boston Medical Center, Boston, Massachusetts

**Keywords:** INH-resistant tuberculosis, pleural tuberculosis, tuberculosis

## Abstract

Drug-resistant tuberculosis (DR-TB) remains a major public health threat. A 23-year-old man presented with fever, dyspnea, and a pleural effusion. After a delay, he was diagnosed with isoniazid (INH)-resistant TB. We review the literature describing the epidemiological and clinical significance of INH-resistant TB and its relevance for low-incidence countries, such as the United States.

Drug-resistant tuberculosis (DR-TB) remains one of the greatest public health challenges of the 21^st^ century [1]. Isoniazid monoresistance (INH-R) is estimated to occur in 8% of TB cases worldwide and is associated with worse treatment outcomes [2], but it often remains undiagnosed or diagnosed after significant delays [3]. We describe a patient with a delayed diagnosis of INH-resistant TB and review the literature on its clinical significance and management approach.

## CASE REPORT

**Figure 1.**
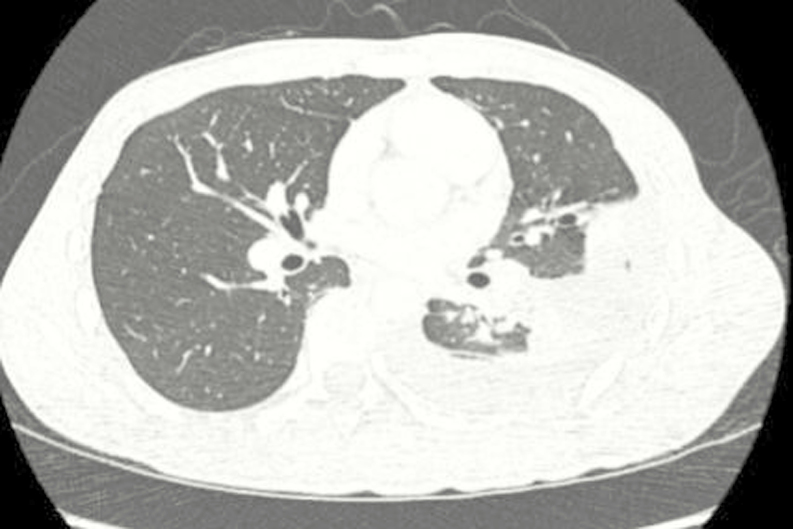
Chest CT demonstrated a moderately sized left-sided, loculated pleural effusion tracking along the major fissure without a rim of pleural enhancement to suggest empyema.

A 23-year-old HIV-negative man presented with 4 days of fever, dyspnea, and pleuritic chest pain. He was previously healthy. He had immigrated from Vietnam 4 months prior. Computed tomography of the chest confirmed a left-sided pleural effusion without evidence of empyema (Figure 1).

Thoracentesis revealed a lymphocytic predominant exudative effusion with mildly elevated adenosine deaminase. Acid-fast bacilli (AFB)-induced sputum smears were negative, but Xpert MTB/RIF nucleic acid amplification testing was positive for *Mycobacterium tuberculosis* without rifampin resistance. He was started on therapy with rifampin, isoniazid, ethambutol, and pyrazinamide (RHZE) and discharged home.

Approximately 9 weeks later, final sensitivity results of the AFB sputum and pleural fluid culture returned, showing *Mtb* with resistance to INH at drug concentrations of 0.2 and 1.0 mcg/ml and susceptibility at 5.0 mcg/ml (agar proportion method, solid media). This was thought to represent low-level INH resistance. At this point, the patient had already completed 9 weeks of therapy, and he was clinically much improved with resolution of presenting symptoms and dramatic improvement in his imaging. He completed 6 months of therapy with RHZE and remained clinically well at a 3-month posttreatment follow-up visit.

## EPIDEMIOLOGY OF INH RESISTANCE

In Vietnam, approximately 4% of new cases of TB are multi-drug resistant (MDR) [1]. According to a 2011 national drug resistance survey in Vietnam, 5% of new TB patients with available susceptibility data were INH monoresistant, compared with 10.4% of previously treated patients. In addition, 18.9% of all new cases and 44.7% of previously treated cases had INH resistance [4]. A 2013 study of newly diagnosed TB patients in Hanoi, the capital of Vietnam, found that any INH resistance was observed in 28.2% of isolates, with 10.0% of isolates having INH monoresistance, suggesting INH-R TB may be more prevalent in urban areas [5].

Globally, it is estimated that 16.1% of TB disease cases in the former Soviet Union and 7.5% of cases outside of these settings have INH monoresistance [6]. In the U.S., the prevalence of INH monoresistance was approximately 9.3% of cases in 2017, having increased from 8.4% in 2006 [7]. The rate of INH monoresistance has risen slowly and steadily for several years. The rate of INH monoresistance varies by state, and INH resistance is more prevalent in foreign-born than in American-born people [8].

## DIAGNOSIS OF INH RESISTANCE

Traditionally, INH-resistant TB is diagnosed via phenotypic drug-susceptibility testing (DST). Culture can be performed using solid media or automated liquid broth culture systems. Culture-based DST allows for comparison of growth on a drug-containing medium with a control medium to establish the presence of drug resistance. The level of drug in the culture medium that inhibits 95% of wild-type TB strains, which have not been exposed to the drug but do not suppress the growth of strains that are resistant to the drug, is known as the “critical concentration” [9]. INH is routinely tested at more than one concentration. If the drug is found to be resistant at a lower concentration but susceptible at a higher concentration, it may still be used in a treatment regimen if it is possible to achieve sufficiently high serum-drug concentrations.

Resistance to INH occurs via mutations to the katG or inhA genes 85–90% of the time, according to surveillance data [10, 11]. However, other mutations, including those in the ahpC– oxyR intergenic region, can confer INH resistance [12] and, thus, phenotypic DST may play a continued role if molecular tests for resistance are restricted to katG and inhA.

KatG mutations are thought to lead to high-level INH resistance, making INH ineffective for the treatment of *Mycobacterium tuberculosis* with this mutation. Low-level resistance to INH (inhA mutated) signifies that high doses of INH may still be effective against *M. tuberculosis* [13, 14].

The Xpert MTB/RIF and Ultra assays do not detect mutations in *katG* and *inhA*. Because Xpert is the initial recommended test, INH-R TB will usually be missed until final drug susceptibility results return (assuming culture has been obtained), months into therapy.

The MTBDRplus test is capable of detecting INH resistance, with 90% sensitivity and 99% specificity, and may reduce the time to the initiation of treatment for MDR-TB [15]. However, this assay is less widely used because it is technically more complex to perform and requires significant laboratory infrastructure.

A recent study evaluated an automated cartridge-based molecular assay and demonstrated sensitivity of 83.3% for INH-R compared to phenotypic DST, which rose to 98.1% when compared to DNA sequencing [16]. However, this test is designed to be used after Xpert MTB/RIF or Ultra demonstrates RIF resistance and, thus, may not contribute significantly to the diagnosis of INH monoresistance. There has been some other progress in the diagnostic pipeline for tests that may enable rapid detection of isoniazid resistance, including the BD MAX MDR-TB assay, which has a CE mark and is available in Europe, although available clinical data are limited and this would require reference laboratory infrastructure [17]. In the U.S., the molecular detection of drug resistance (MDDR) assay, which uses targeted sequencing, can be performed at the Centers for Disease Control and Prevention to detect drug resistance to first and second line drugs, including INH, and is available to clinicians if patients meet criteria for testing for drug-resistant TB [10].

## TREATMENT OF INH MONORESISTANCE

The current U.S. guidance for INH-R TB (from the Curry International Tuberculosis Center) is based largely on expert opinion informed by retrospective or single-arm studies [18]. Three options for known INH-R TB are usually recommended. First is daily rifampin, ethambutol, or pyrazinamide combined with a fluoroquinolone (such as moxifloxacin or levofloxacin). After 2 months, the regimen may be narrowed to rifampin, moxifloxacin, and ethambutol or to just rifampin and moxifloxacin. Second, if the patient does not tolerate pyrazinamide, a regimen consisting of rifampin, ethambutol, and a fluoroquinolone for 9 to 12 months may be used. Third is rifampin, pyrazinamide, and ethambutol and a fluoroquinolone for 2 months, followed by once weekly high-dose rifapentine and moxifloxacin for 4 months, based on the RIFAQUIN trial [19].

In contrast, the updated WHO 2018 guideline recommends using rifampin, pyrazinamide, ethambutol, and levofloxacin for 6 months in patients with confirmed rifampin-susceptible INH-R TB [3]. A retrospective cohort study from South Korea demonstrated that unfavorable outcomes, including treatment failure and relapse were higher amongst patients who did not receive fluoroquinolones (8.8% compared to 1.5%, *P* = .037) [20]. A 2018 meta-analysis found an advantage to adding a fluoroquinolone to the daily rifampin, ethambutol, and pyrazinamide regimen with moderately higher odds of treatment success (adjusted odds ratio = 2.8) [21]. However, fluoroquinolone use had no significant effect on mortality or the acquisition of rifampin resistance. Of note, the WHO guideline recommends levofloxacin due to the interaction with rifampin (ie, reduced bioavailability of moxifloxacin).

A 2017 systematic review estimated that 15% of patients with unrecognized INH-R TB treated with a range of regimens, including first-line therapy, had a combined outcome of treatment failure, relapse, or both [2]. Another review demonstrated that extending the duration of RIF and increasing the number of effective drugs at 4 months was associated with a lower odds of treatment failure, relapse, and death [22]. However, there have been no randomized controlled trials (RCTs) evaluating regimens for INH-R TB, and RCTs evaluating shorter regimens for drug susceptible TB have not demonstrated superiority of fluoroquinolone-based regimens for patients with INH-R TB [19].

The role of isoniazid at standard or high-doses (10-15mg/kg/day) within the regimen for INH-R TB has not been established, although there is in vitro evidence to suggest that it may be effective in patients with low-level resistance, such as ours [23]. However, in such cases, isoniazid is not typically counted as a fully active drug in the regimen. After treatment is completed, ongoing monitoring should be performed in patients with INH-R TB. Higher minimum inhibitory concentration (MIC) values of isoniazid and rifampin have been associated with a greater risk of relapse of TB than lower MIC values [24].

In our case, a fluoroquinolone was not included once INH resistance was determined, because the patient had already improved clinically and was not thought to have a high burden of disease that would warrant the addition of fluoroquinolone per the Curry Center guidelines.

## CONCLUSION

Without rapid testing for INH resistance, the appropriate implementation of a reliably effective regimen can be delayed, as seen in this case. This has individual- and population-based implications, as INH-R is associated with higher treatment failure and relapse rates and as these strains may evolve into DR-TB strains [25]. Clinicians should consider the possibility of INH-R TB in patients from high incidence settings, use available assays such as MTBDRplus or MDDR where possible, and consider a fluoroquinolone-based regimen once INH-R is confirmed.
